# First report of community dynamics of arbuscular mycorrhizal fungi in radiocesium degradation lands after the Fukushima-Daiichi Nuclear disaster in Japan

**DOI:** 10.1038/s41598-019-44665-7

**Published:** 2019-06-03

**Authors:** Masao Higo, Dong-Jin Kang, Katsunori Isobe

**Affiliations:** 1 Department of Agricultural Bioscience, College of Bioresource Sciences, Nihon University, Kameino 1866, 252-0880 Fujisawa, Kanagawa Japan; 2 Teaching and Research Center for Bio-coexistence, Faculty of Agriculture and Life Science, Hirosaki University, 037-0202 Gosyogawara, Aomori Japan

**Keywords:** Arbuscular mycorrhiza, Microbial ecology

## Abstract

Arbuscular mycorrhizal fungi (AMF) can be beneficial for improving restoration of radioactive-cesium (^137^Cs)-contaminated soils through soil remediation. However, there has been no information on species diversity and the composition of AMF communities in ^137^Cs-contaminated soil after the Fukushima-Daiichi Nuclear Power Plant (NPP) disaster. We examined the community dynamics of indigenous AMF colonizing roots of napiergrass (*Pennisetum purpureum*) in two different ^137^Cs-contaminated land-use fields (grassland and paddy field) by an Illumina MiSeq sequencing investigation within a 30-km radius around the Fukushima-Daiichi NPP in 2013 (sampling year 1) and 2014 (sampling year 2). We found nine AMF families, including Glomeraceae, Gigasporaceae, Paraglomeraceae, Claroideoglomeraceae, Acaulosporaceae, Archeosporaceae, Ambisporaceae, Diversisporaceae and uncultured Glomeromycotina in roots. Glomeraceae was the most abundant in both grassland and paddy field, followed by Paraglomeraceae. The diversity of AMF in grassland and paddy field was higher in 2014 than in 2013. Furthermore, the AMF community structure was impacted by sampling year and land-use type. The AMF community structures colonizing napiergrass roots were also significantly impacted by land-use type and year throughout the 2-year investigation. To our knowledge, our results are the first report to reveal the community dynamics of indigenous AMF in the ^137^Cs-contaminated fields around NPP.

## Introduction

A catastrophic earthquake and tsunami occurred in Japan on March 11, 2011, which severely damaged the Fukushima-Daiichi Nuclear Power Plant (NPP). The disaster also triggered the worst nuclear accident in world history since Chernobyl. This disaster led to emissions of radioactive materials such as, radiocesium (^137^Cs and ^134^Cs), strontium-90 (^90^Sr), iodine-131 (^131^I), tellurium-132 (^132^Te) and xenon-133 (^133^Xe), from the NPP^1^. Among these materials, ^137^Cs especially provokes the largest concern because of its devastating effect on agriculture and stock farming for decades. For ^137^Cs, a total emission of 36.6 (20.1–53.1) PBq was released into the atmosphere during the NPP disaster^2^, and this contaminated the soil, over a vast area. In the current ^137^Cs-removal approach at Fukushima Daiichi NPP, Kang *et al*.^3,4^ reported that napiergrass (*Pennisetum purpureum* Schum.), which has a large aboveground biomass and a high ability to accumulate Cs in its shoots, as a highly suitable potential phytoremediation crop^3–5^. Addtionally, the same author suggested the possibility of increasing Cs transfer from the Cs-contaminated soil around the NPP using napiergrass^3^.

Arbuscular mycorrhizal fungi (AMF) can increase host plant phosphorus (P) uptake and growth, and AMF may especially improve plant micronutrients uptake^6^. Additionally, the role of AMF in radionuclide-contaminated soils and their effects on radioactive-Cs uptake and transfer to plants have been examined with a possible way to develop the use of AMF for soil remediation strategies^7–9^. Hammer *et al*.^10^ indicated that AMF selectively absorb potassium (K) which is an analog for Cs. In addition, Cs is a weak analog of K, but is a better analog of rubidium (Rb). These analogs are accumulated by fruiting bodies of mushrooms, such as saprotrophic and ectomycorrhizal fungi^11^. Previous studies have shown no correlation between ^137^Cs and K uptake in mushrooms^12^. On the contrary, ^137^Cs and ^133^Cs uptake have been positively correlated with Rb uptake in fruiting bodies of mushrooms, such as saprotrophic and ectomycorrhizal fungi^11^. Likewise, AMF can also take up, accumulate, and translocate ^137^Cs in their external hyphae^13,14^. These results indicate a potential involvement of AMF in the Cs biogeochemical cycle and in plant Cs accumulation. However, previous studies have also demonstrated that AMF either facilitate, or have no effect on ^137^Cs uptake by host plants. Thus, the actual role of AMF in plant uptake of Cs, and the capacity and AMF species to accumulate is not fully understood and remains challenging because there is no ecological and functional information of the AMF in ^137^Cs-contaminated soil due to the Fukushima-Daiichi NPP accident.

Furthermore, AMF community structure as well as nutritional improvement by AMF can play an important role in ecosystem restoration and sustainability^15,16^. Many studies have reported AMF communities can impact a number of important ecosystem processes, including plant productivity, plant diversity and soil structure^17,18^. To promote and evaluate efficient characterization of AMF communities by sequencing techniques, current studies have relied on next-generation sequencing technologies, such as Illumina Miseq platform^19,20^. Therefore, understanding AMF diversity and communities can be important to explore the ecological environment^21^.

Considering the above facts, combining napiergrass as a candidate plant with high potential for ^137^Cs-remediation and the ^137^Cs uptake function by AMF in the ^137^Cs-contaminated soil in Fukushima may be a better technique to remove ^137^Cs from the ^137^Cs-contaminated soil. However, the role of AMF combined with napiergrass in processes of ^137^Cs uptake are unclear and not fully understood. Therefore, we investigated the distribution of indigenous AMF colonizing roots of napiergrass in the ^137^Cs-contaminated soil around the NPP as a preliminary investigation. This study was undertaken as a first investigation of the distribution of indigenous AMF colonizing roots of napiergrass that grew in two high ^137^Cs-contaminated soils within a 30-km radius around the Fukushima-Daiichi NPP. Additionally, the indigenous AMF communities in roots of naipergrass from ^137^Cs-contaminated soils were investigated in detail using a next-generation sequencing of the Illumina Miseq platform for the first time.

## Materials and Methods

### Experimental design

We conducted a field study with two objectives. First objective was to understand whether different plant densities in naipergrass increase the ^137^Cs removal from the ^137^Cs-contaminated soil based on a study by Kang *et al*.^3^. Second objective according to our main purpose of the study was to determine the distribution of indigenous AMF colonizing roots of napiergrass that grew in two high ^137^Cs-contaminated soils within a 30-km radius around the Fukushima-Daiichi NPP. Thus, a field experiment was conducted in two different land-use types (lowland and upland soils) in Fukushima, Japan. This experiment was performed in collaboration with Namie town and with the permission of the Ministry of Environment, Japan. Two experimental sites were used based on their different land-use histories before the NPP accident. Both the lowland and upland fields were used as a paddy field (37.486381N, 140.959085E) and grassland (37.489980N, 140.949368E), respectively, before the disaster of the Fukushima-Daiichi NPP on March 11, 2011. Physicochemical properties of the paddy field and grassland at the beginning of the field experiment in 2013 are shown in Table S1, which were adapted from Kang *et al*.^3^. Further details on the methods of soil physicochemical properties regarding each land-use type are presented in Kang *et al*.^3^.

To determie the communities of AMF colonizing roots of napiergrass (*P*.*purpureum* Schum., var. Merkeron), three of the 2.0 m × 2.0 m plots were established in both the paddy field and grassland. The planting density in plot for napiergrass at each land-use type were low density (4 plants plot^−1^), medium density (16 plants plot^−1^), and high density (44 plants plot^−1^), which were randomly distributed. Four-week-old napiergrass nursery plants were transplanted into plots with 0.3-m (high density), 0.5-m (medium density) or 1-m (low density) interrow spacing between each plot on May 27, 2013 and on May 17, 2014. The amount of N- and P-applied rates were 10 and 10 g m^−2^, respectively. No K fertilizer was applied to the two fields during this 2-year investigation. Further details on the information of plant growth parameters and uptake of Cs regarding each land-use type are presented in Kang *et al*.^3^.

### Root sampling and staining

Root samples in napiergrass were manually collected from three plants in each plot (to a depth of 15 cm, with a diameter of 20 cm), resulting in three independent root samples products per plot. A total of nine independent root samples of napiergrass in each land-use type (a total of 18 root samples in each year) were analyzed for morphological traits and for DNA extraction on October 29, 2013 (year 1), and October 28, 2014 (year 2). The root samples were stained with 5% (w/v) black ink-vinegar solution^22^, and the AMF root colonization was measured according to Giovannetti and Mosse^23^.

### DNA extraction and PCR amplification

Genomic DNA was taken from 50 mg of fresh root samples using the DNeasy Plant Mini Kit (Qiagen, GmbH, Germany) according to the manufacturer’s instructions. The DNA pellet was resuspended in 50 μl of TE buffer (pH 8.0) and stored at −30 °C until use for polymerase chain reaction (PCR). In total, 36 samples from the grassland field and a paddy field in the 2-year investigation were subjected to DNA extraction. The DNA samples extracted from roots were used as PCR templates. To decrease variations in the PCR process, samples were amplified in triplicate^24^ using the fusion primer set in a PCR at 10 μl per subsample, resulting in three independent PCR products per plant. Primers AMV4.5NF (forward) and AMDGR (reverse)^25^ were used for the amplification of AMF 18 S rRNA gene fragments by Mastercycler ep gradient (Eppendorf, Hamburg, Germany). PCR was performed in 10 μl reaction mixtures containing each 2X of reaction buffer, 0.3 μM of forward and reverse primers (10 μM), 1 U of Taq DNA polymerase (KOD multi & Epi, Toyobo, Japan), and 1 µl of template DNA. The PCR protocol was composed of initial treatment at 94 °C for 2 min; 45 cycles of treatments at 98 °C for 10 s and at 60 °C for 10 s.

### Molecular diversity of AMF communities in roots

To reduce potential early-round PCR errors, three independent PCR products per plant were pooled together and purified using NucleoSpin Gel and PCR Clean-up kit (Macherey-Nagel, Duren, Germany), and quantified using UV spectrophotometry (DS-11 NanoPad, DeNovix Inc., USA). The purified PCR amplicons were normalized before pyrosequencing. The purified amplicons were paired-end (PE) sequenced on an Illumina MiSeq platform (Bioengineering Lab Co., Ltd, Kanagawa, Japan). In total, 36 sequencing libraries were constructed and independently sequenced. Sequence read processing was performed using QIIME version 1.9.1^26^.

The reads were truncated at any site that received an average quality score <20 over a 40 bp sliding window, and the truncated reads shorter than 40 bp were discarded using FASTX-Toolkit. Then, PE reads were assembled according to their overlap sequence with a minimum overlap length of 10 bp, while reads that could not be assembled were discarded. The clean sequences were analyzed using the FLASH (Fast length adjustment of short reads). Chimeric sequences were identified and removed using UCHIME in USEARCH. Representative sequences were checked against the Maarj*AM* AMF database^27^ and NCBI GenBank. Phylogenetic analyses were performed using the Neighbor-joining (NJ) phylogenic trees (Tamure-Nei model) and maximum likelihood (TN93 + G) algorithms implemented in the program MEGA 7.0^28^. Bootstrap values were estimated from 1,000 replicates. Additionally, the AMF OTU groups were classified according to Redecker *et al*.^29^. Representative sequence OTUs, defined as groups of closely related sequences with a high level of bootstrap support in the phylogenetic analysis, were selected. If more than one sequence from our study was present in the same OTU cluster, one was chosen as the representative sequence. Following the same similar approach as in Sosa-Hernández *et al*.^30^, we considered matches with ≥97% similarity a species level match, ≥90% a genus level match, and ≥80% a family level match. A species level match refers to how confidently we assigned a name to the OTU based on known sequences, and did not imply that these OTUs are to be considered equivalent to those species. The raw sequence data are available in the the DNA Data Bank of Japan (DDBJ) (DDBJ Sequence Read Archive, DRA006649, BioProject Accession: SSUB009022). Additionally, we calculated measures of sample coverage for all taxa using the R package *vegan*.

### Statistical analysis

An arcsine-square root transformation was used for normalization of the data of AMF colonization in the roots of napiergrass. We determined significant differences between land-use types and sampling years were assessed using two-way analysis of variance (two-way ANOVA). In addition, Hill numbers (or the effective number of species) have been increasingly used to quantify the species/taxonomic diversity of an assemblage because they represent an intuitive and statistically rigorous alternative to other diversity indices^31^. Thus, diversity of AMF OTU communities were measured based on the first three Hill numbers, such as species richness, Shannon diversity (the exponential of Shannon entropy), and Simpson diversity (the inverse Simpson concentration) using the package *iNEXT* in R 3.4.2 (http://www.r-project.org/)^31^. The AMF communities among land-use types and sampling years were assessed using venn diagram analysis with the R package *gplots*.

We conducted permutational multivariate analysis of variance (PERMANOVA) using the *vegan* package in R 3.4.2 to examine the effect of land-use type and sampling year on AMF community structure^32^. To determine the relationship of land-use type and sampling year with respect to AMF communities, we performed redundancy analysis (RDA) as a multivariate analysis using the package *vegan* in R 3.4.2. The data of AMF communities were log-transformed. The sequences data matrix was composed of the abundance of AMF families and land-use type or sampling year. The environmental variable of land-use type and sampling year on the AMF communities were estimated according to Higo *et al*.^33,34^. This analysis was carried out using the *vegan* package in R 3.4.2 with 999 permutations of the Monte-Carlo test. To investigate if AMF community structure differed significantly between land-use type and sampling year, the PERMANOVA was performed with 9999 permutations using the *adonis* function in the package *vegan* in R 3.4.2.

## Results

### AMF colonization in the two different land-use types

**Figure 1:**
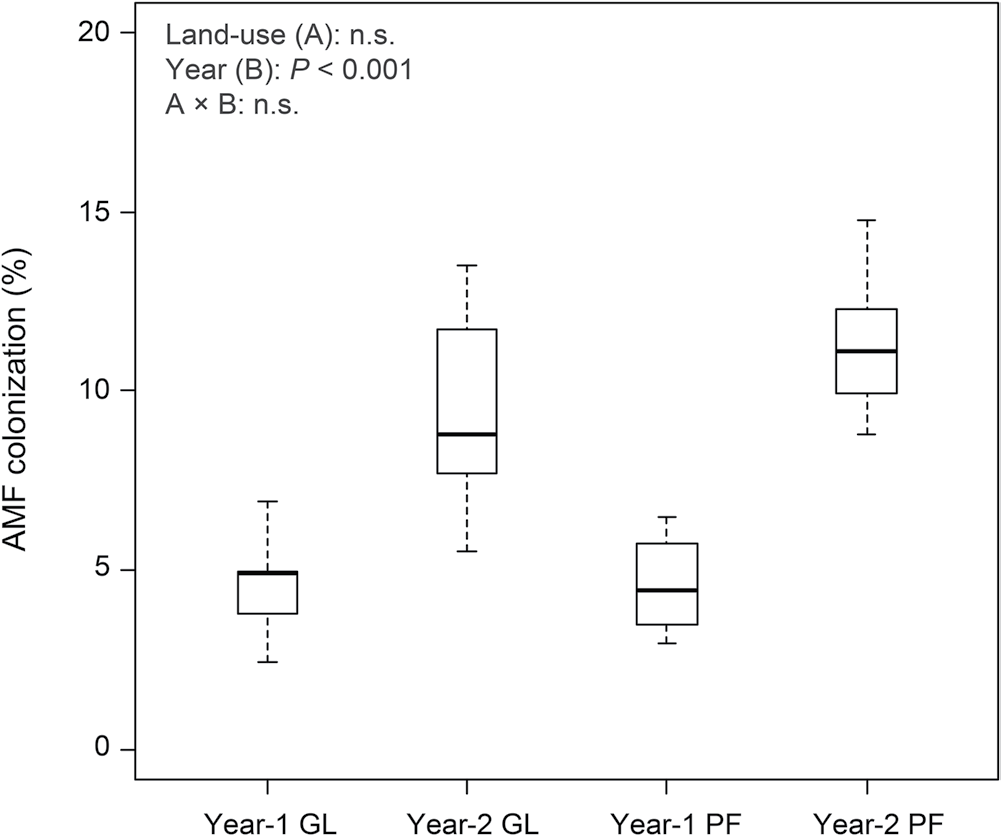
Boxplots illustrating differences in group averages of root colonization of arbuscular mycorrhizal fungi (AMF) in napiergrass at two land-use types in sampling years 1 and 2. Bold horizontal lines represent median values; box margins ± SE and vertical lines represent minimum and maximum values of the groups. Error bars indicate standard errors of the means (n = 9). GL = grassland, PF = paddy field.

Overall, AMF colonization in the napiergrass regardless of land-use type was never greater than 20% throughout the 2-year field study (Fig. 1). The AMF colonization ranged from 3.9% to 6.0% in grassland and from 4.5% to 4.7% in paddy field in sampling year 1 (2013). In sampling year 2 (2014), the AMF colonization ranged from 8.7% to 10.8% in the grassland, and from 10.8% to 11.7% in the paddy field. The AMF colonization in the napiergrass was influenced by sampling year, but was not influenced by land-use type.

### General sequencing information and taxonomic richness

In this study, a total of 3,861,816 paired-end sequences were obtained from the 36 libraries using the AMV4.5NF/AMDGR primer set. Of these, 3,264,994 sequences belonged to Glomeromycotina (corresponding to 84.5% of the total). We showed the taxonomic distributions of the obtained sequences in root samples (Table S2). We found a total of 230 OTUs in the AMF communities based on AMF family level (Table S2). The phylogenetic placement of OTUs in different land-use types was determined using the phylogenetic tree. The AMF OTUs were classified into one of nine AMF families including Glomeraceae (average relative abundance: 39.97%), Gigasporaceae (22.03%), Paraglomeraceae (33.06%), Claroideoglomeraceae (1.32%), Acaulosporaceae (1.06%), Archeosporaceae (0.90%), Ambisporaceae (0.38%), Diversisporaceae (0.18%) and unknown Glomeromycotina in roots (4.37%) (Fig. S1).

### Diversity of AMF communities in the two different land-use types

**Figure 2:**
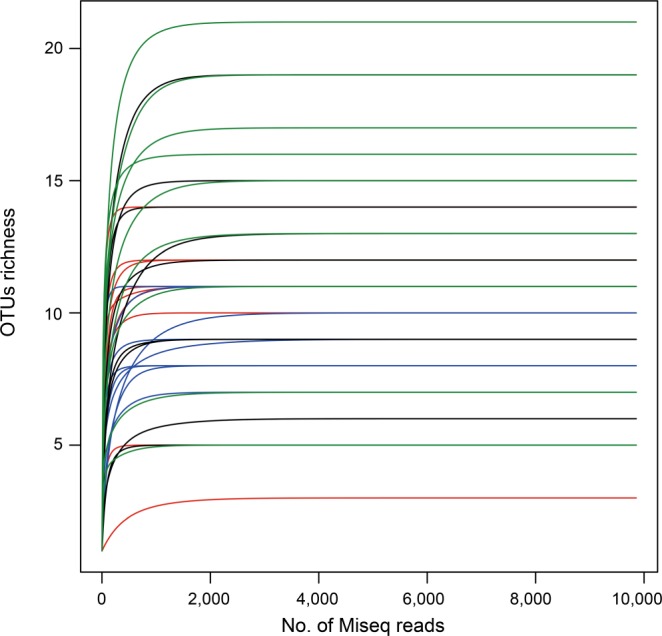
Rarefaction curves showing the sequencing depths in the napiergrass roots in this study. Sampling year-1 grassland: red lines, sampling year-1 paddy field: blue lines, sampling year-2 grassland: black lines, sampling year-2 paddy field: green lines.

**Figure 3:**
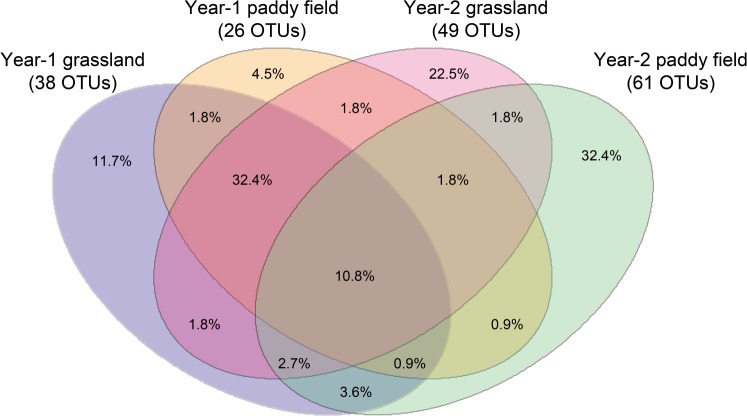
Venn diagram showing the operational taxonomic unit (OTU) overlap among different land-use types around the Fukushima-Daiichi Nuclear Power Plant (NPP). Numbers in parentheses indicate total OTU in each land-use type, and numbers inside the venn diagram indicate unique and shared OTUs.

**Figure 4:**
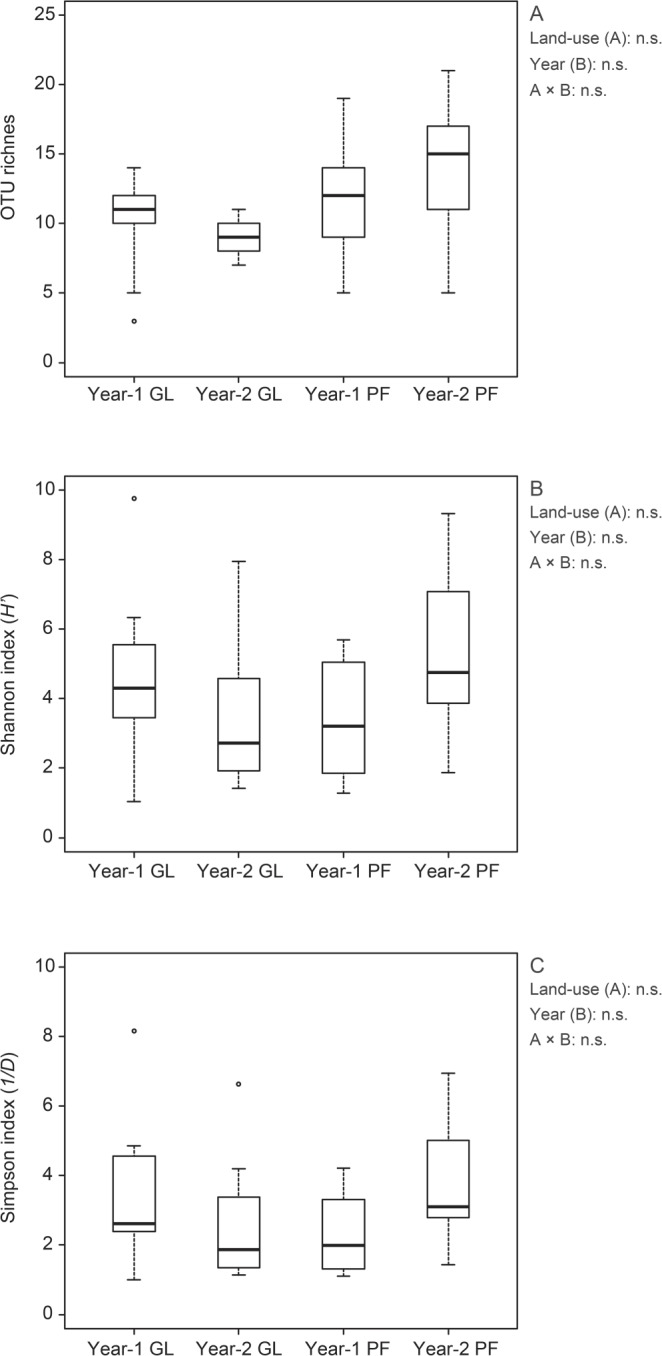
Boxplots illustrating differences in group averages regarding (**A**) operational taxonomic unit (OTU) richness, (**B**) Shannon index (*H*′) and (**C**) Simpson index (*1/D*) in the roots of napiergrass at two land-use management types in sampling years 1 and 2. Bold horizontal lines represent median values; box margins ± SE and vertical lines represent minimum and maximum values of the groups. n.s. = not significant by two-way ANOVA. Error bars indicate standard errors of the means (n = 9). GL = grassland, PF = paddy field. Circles show outliers.

A plateau was reached by all rarefaction curves (Fig. 2), which shows that high enough sequenced reads could identify most sequence types at a 97% similarity level (Table S3). The samples from roots in the sampling year 2 (2014), regardless of land-use type, had a larger number of OTUs (49–61) compared with those from roots in sampling year 1 (26–38OTUs). Additionally, we found 38, 26, 49 and 61 AMF OTUs in the sampling year-1 grassland, year-1 paddy field, year-2 grassland and year-2 paddy field, respectively (Fig. 3). The number of total OTUs in this experiment was 111, of which only 10.8% OTUs were common in all treatments. OTUs that occurred specifically in only the sampling year-1 grassland, year-1 paddy field, year-2 grassland and year-2 paddy field land-use types were 11.7%, 4.5%, 22.5% and 32.4%, respectively. In addition, the richness of root OTUs from the paddy field in sampling year 2 had a larger number of OTUs compared with those from the paddy field in sampling year 1 (Fig. 4A). No similar tendency in the OTU richness, Shannon diversity (*H*′) and Simpson index (*1/D*) was found in grassland. Additionally, no significant difference in the OTU richness between paddy field and grassland was found in both sampling years (Fig. 4A–C).

### Dynamics of AMF communities in the two different land-use types

**Figure 5:**
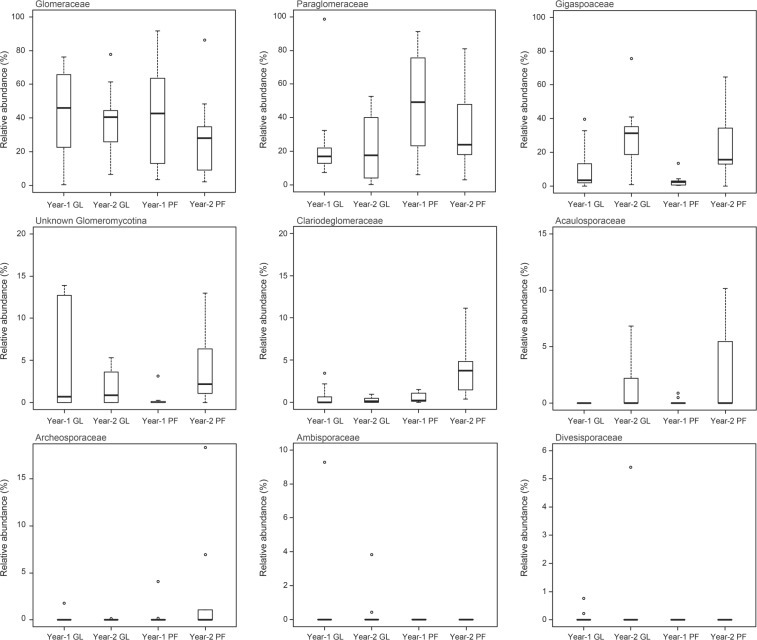
Boxplots illustrating differences in group averages of each family of arbuscular mycorrhizal fungi (AMF) in napiergrass roots at two land-use management in the sampling years 1 and 2. Bold horizontal lines represent median values; box margins ± SE and vertical lines represent minimum and maximum values of the groups. GL = grassland, PF = paddy field. Circles show outliers.

Land-use type and sampling year strongly impacted AMF communities by influencing the proportions of three dominant AMF families (Fig. 5). Glomeraceae was predominant and detected at a much higher frequency in both grassland and paddy field regardless of sampling year. Gigasporaceae was more abundant in sampling year 2 compared with in sampling year 1 regardless of land-use type, and Paraglomeraceae in the paddy field in sampling year 2 had a larger number of abundance compared with in sampling year 1. No similar tendency was found in grassland. The distribution of each AMF family was different in each land-use type during the 2-year experiment.

### Dynamics of AMF communities in the two different land-use types

**Figure 6:**
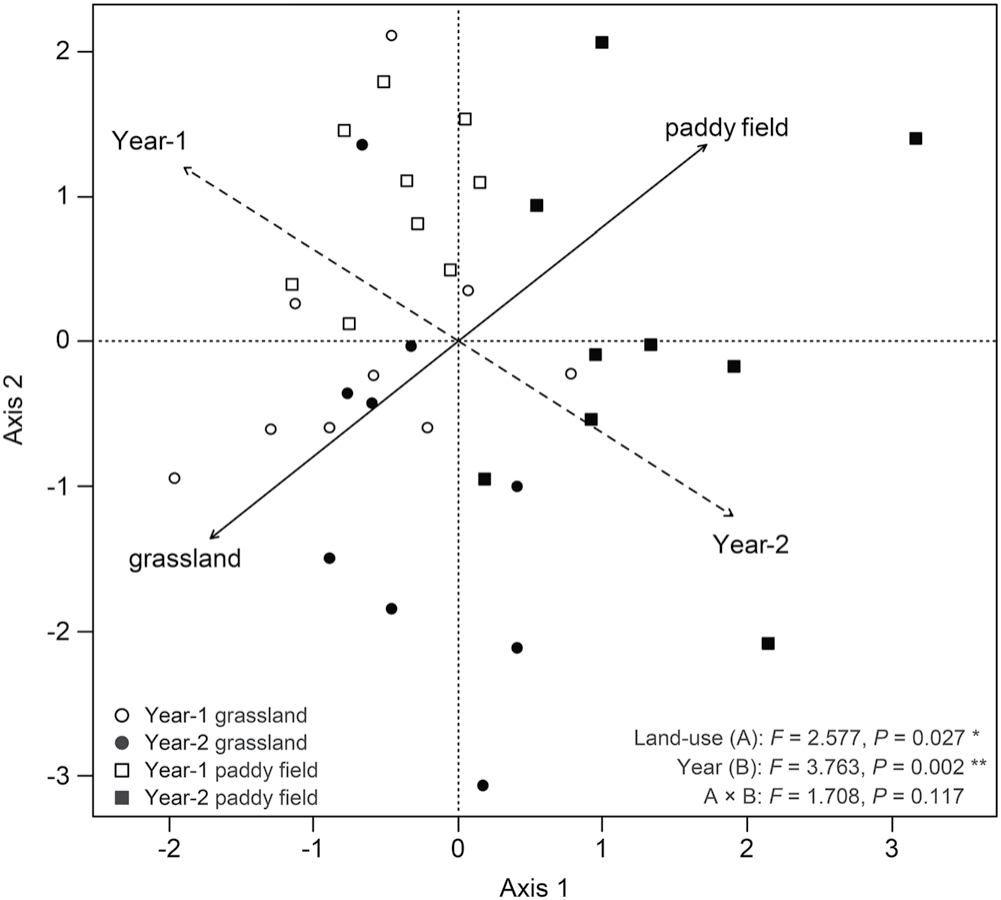
Redundancy analysis (RDA) of communities of arbuscular mycorrhizal fungi (AMF) in roots sampled from two land-use types and sampling year. The environmental variables explain 18.4% and 14.6% of the total variations in the first two axes. Variables with significant effects in Monte Carlo tests (*P* < 0.05) are shown. Solid lines indicate effects of land-use types, and dashed lines indicate the effects of sampling year.

The RDA trends clearly showed that the land-use type and sampling year significantly altered the AMF community structure in the roots (Fig. 6). As shown in Fig. 6, the AMF communities in the sampling year-2 plots were in the first (paddy field) and fourth (grassland and paddy field) quadrants, while most of the AMF communities in the sampling year-1 plots were in the second (paddy field) and third (grassland) quadrants. The ordination diagram indicates that paddy field and grassland (*R*^2^ = 0.756, *P* = 0.001), and the sampling year-1 and year-2 plots (*R*^2^ = 0.801, *P* = 0.001) contributed significantly to the variation in AMF root communities (Fig. 6). We also conducted PERMANOVA to determine the effect of each land-use type and sampling year to the AMF root communities. The result of PERMANOVA showed that land-use type (*F* = 2.577, *P* = 0.027) and sampling year (*F* = 3.763, *P* = 0.002) significantly affected the AMF root community structure, but the AMF root communities in the roots were not affected by the interaction of land-use and sampling year (*F* = 1.708, *P* = 0.1117) (Fig. 6).

## Discussion

Our study is the first investigation to reveal the distribution of indigenous AMF colonization and communities in the agricultural field around the Fukushima-Daiichi NPP after the March 11 disaster.

### Occurrence of AMF colonization in roots

We observed the AMF colonization of napiergrass in Cs-contaminated soil (Fig. 1), which had slightly lower AMF colonization than that observed in other studies regardless of land-use type and sampling year^7,35^. A previous study reported that AMF root colonization in plants was negatively affected by increased soil Cs concentrations^36^, in agreement with the findings of our study. Thus, higher soil ^137^Cs concentration may inhibit the activity of the indigenous AMF population in the soil to promote mycorrhization in the ^137^Cs-contaminated soil. On the contrary, others studies have shown that soil Cs concentration did not inhibit AMF root colonization of leek, ryegrass, barely, cucumber, and sunflower^7,35^. Furthermore, it is unclear whether napiergrass increased Cs uptake from Cs-contaminated soil in terms of AMF root colonization. This is because our study did not examine the transfer of Cs uptake by napiergrass with AMF. However, Gyuricza *et al*.^8^ reported the capacity of AMF to transport radioactive Cs under *in vitro* conditions. These authors demonstrated that AMF could take up, translocate and transfer radioactive Cs to their host through their extraradical hyphal network. Thus, further investigation into the functional aspects of AMF root colonization on ^137^Cs uptake would provide support to elucidating the benefit of AMF on ^137^Cs removal from Cs-contaminated soil around the Fukushima-Daiichi NPP.

### Distribution of AMF taxa in roots

Here, for the first time, we used an Illumina Miseq Platform of an approximately 250 bp SSU rDNA amplicon together with a high throughput phylogenetic annotation method to investigate AMF taxa in the ^137^Cs-contaminated soil. Overall, the most frequently detected families or OTUs in the roots of napiergrass between land-use type and sampling year belonged to Glomeraceae (Figs 5 and 6), which are commonly found in grasslands and arable lands^37–40^. Glomeraceae are especially predominant among arable fields because they adapt well to disturbed environments compared with other families as well as have high sporulation rates for rapid recovery^41^. Furthermore, previous studies have shown that Glomeraceae including the genus of *Glomus*, *Funneliformis*, *Rhizophagus*, and *Paraglomus* can form mycorrhizae in plant roots via their fragments of mycelium or colonized root fragments, rapidly constructing a anastomosis of hyphae^42^. However, Gigasporaceae including the genus of *Gigaspora*, *Racocetra*, and *Scutellospora* spreads and promotes via the dispersal of spore or infection from an intact mycelium^43,44^. Additionally, some other studies have indicated that Glomeraceae and *Glomus*-like genus (*Paraglomus* genus) have a certain resistance in complex environments^45,46^. As a result, these characteristics help with the survival and spread of *Glomus*-like genus including *Paraglomus* in a Cs-contaminated soil. Furthermore, the differences between Gigasporaceae and Glomeraceae clearly contribute to the dominance of the Glomeraceae OTUs over Gigasporaceae OTUs in roots from each land-use type. However, our results showed that Gigasporaceae OTUs were also relatively abundant among other detected AMF families. This result suggests that the emergence of the taxa can result from adaptation to the Cs-contaminated ecological environment.

Furthermore, previous studies Oehl *et al*.^47^ and Alguacil *et al*.^48^ reported that soil type strongly influenced AMF community structure as well as the prevalence and presence of many AMF. The AMF community structures differed between the soil characteristics of paddy field and that of grassland (Figs 5 and 6). Zhao *et al*.^49^ showed that AMF communities were highly affected by soil texture distribution, and their results indicated a significant relationship between the AMF community and the content of silt and sand. In this study, the soil characteristics such as the content of silt and sand were different between the soil of paddy field and that of grassland. These differences in content, and therefore, differences in aeration may be important for plant root growth and soil humus decomposition. Future studies examining the functional aspects of Glomeraceae or Gigasporaceae, and the impact of soil texture and plant root growth would help to elucidate the advantages of specific AMF on ^137^Cs uptake by AMF to plants in ^137^Cs-contaminated soils.

### Dynamics of AMF communities in roots

Year-round shifts in composition of AMF communities in soils and colonizing roots have been reported among diverse plant species under various environmental conditions^33,50–54^. The year-round dynamics of AMF communities showed different structures between land-use type and sampling year (Figs 5 and 6). Similar results have been obtained in some other studies^55–58^. Some AMF OTUs were either replaced or disappeared in the 2-year investigation (Fig. 5). These shifts in OTU abundance and distribution, however, did not lead to changes to overall OTU richness among the AMF communities for either land-use type or sampling year. Thus, longer-term vs short-term trials could determine relationships among land-use type, host crops and AMF. When choosing AMF host crops or land-use type, AMF diversity and abundance should be prioritized to remove ^137^Cs and to ensure a healthy ecosystem in the ^137^Cs-contaminated field around the Fukushima-Daiichi NPP.

## Conclusions

We found that the members of the Glomeraceae, Paraglomerace and Gigasporaceae were predominant in all land-use types. Our results revealed a temporal shift in the AMF communities in the ^137^Cs-contaminated soil around the Fukushima-Daiichi NPP over the 2-year period. Moreover, in different land-use types, diversity and structure of AMF communities also differed. Thus, different land-use types are potentially important to AMF communities. However, it remains unknown whether the AMF communities can be appropriate candidates for the phytoremediation technique for ^137^Cs removal in the long run. Therefore, it is important to better understand the relationships of ^137^Cs in soil and the functions of indigenous AMF communities in terms of ecological aspects to remove ^137^Cs from ^137^Cs-contaminated soil around the area of the Fukushima-Daiichi NPP. This information will provide a better understanding of the interaction between plants and AMF, for future revegetation and phytoremediation studies in polluted sites.

## Supplementary information

Table S1-S3

Supplemental Figure S1
