# Supplementary material for: First report of community dynamics of arbuscular mycorrhizal fungi in radiocesium degradation lands after the Fukushima-Daiichi Nuclear disaster in Japan
Source: Sci Rep. 2019 Jun 3;9:8240. doi: 10.1038/s41598-019-44665-7 (PMC6546702; doi:10.1038/s41598-019-44665-7)
Supplement: Supplementary file 2 — Supplemental Figure S1 [file 41598_2019_44665_MOESM2_ESM.pdf]

Supplementary information

Title: First report of community dynamics of arbuscular mycorrhizal fungi in radiocesium degradation lands after the Fukushima-Daiichi Nuclear disaster in Japan

Masao Higo[1*], Dong-Jin Kang[2], Katsunori Isobe[1]

1 *Department of Agricultural Bioscience, College of Bioresource Sciences, Nihon University, Kameino 1866, Fujisawa, Kanagawa 252-0880, Japan*

2 *Teaching and Research Center for Bio-coexistence, Faculty of Agriculture and Life Sciences, Hirosaki University, Gosyogawara, Aomori 037-0202, Japan*

Corresponding author

Correspondence to Masao Higo

Tel: +81-466-84-3502

E-mail: higo.masao@nihon-u.ac.jp

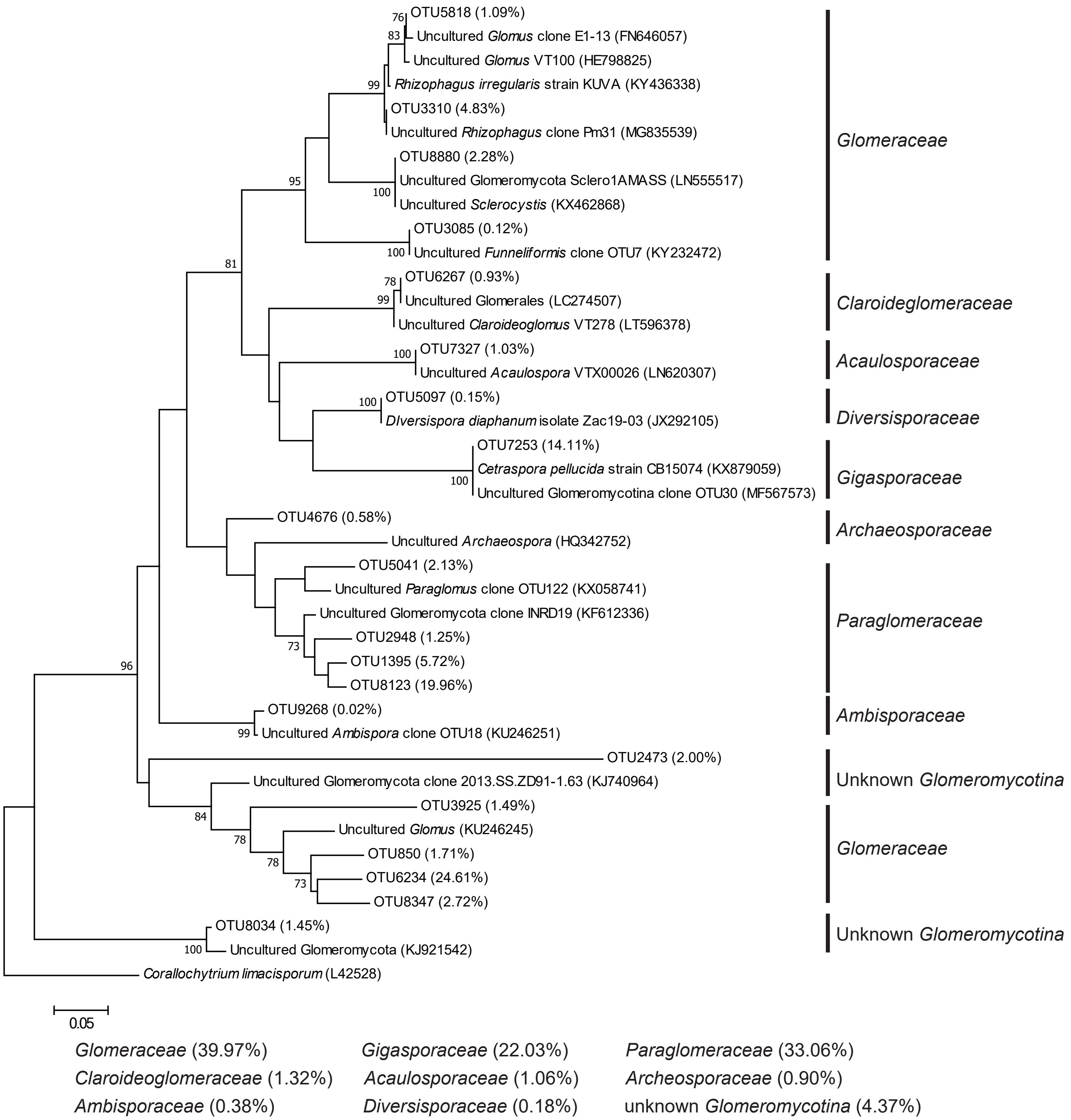

Supplemental Figure S1. A neighbor-joining tree of partial SSU rDNA sequences obtained from the roots of napiergrass based on Tamura and Nei+G substitution model, rooted tree by *Corallochytrium limacisporum* as an outgroup. Bootstrap values (only values > 70 are shown) were estimated from 1,000 replicates. Representative sequences in each AMF family from roots are incorporated in this study. The raw sequence data are available in the DNA Data Bank of Japan (DDBJ) (DDBJ Sequence Read Archive, DRA006649, BioProject Accession: SSUB009022).
